# An inactivated human adenovirus type 55 vaccine attenuates pulmonary pathology in a non-human primate challenge model

**DOI:** 10.1016/j.virusres.2026.199793

**Published:** 2026-08-21

**Authors:** Jun Young Lee, Jung-ah Choi, Dae-Im Jung, Yunjeong Park, Eunji Yang, Chanmi Kim, Minkyung Ko, Seung Ho Baek, Jung Joo Hong, Soon-Hwan Kwon, Won-Tae Kim, Manki Song, Sang Hwan Seo

**Affiliations:** a KeyPrime Research, 193, Osongsaengmyeing 1-ro, Osong-eup, Heungdeok-gu, Cheongju-si, Chungcheongbuk-do, 28161, , Republic of Korea; b Science Unit, International Vaccine Institute, Seoul, 08826, Republic of Korea; c National Primate Research Centre, Korea Research Institute of Bioscience and Biotechnology (KRIBB), Yeongudanji-ro, Ochang-eup, Chengwon-gu, Cheongju, Chungcheongbuk 28116, Republic of Korea; d KRIBB School of Bioscience, Korea University of Science and Technology (UST), Daejeon, Republic of Korea; e Department of Infectious Diseases, Armed Forces Medical Research Institute, Daejeon, Republic of Korea

**Keywords:** Human adenovirus type 55, Acute respiratory disease, Pneumonia, Non-human primate, Cynomolgus macaque

## Abstract

Human adenovirus type 55 (HAdV-55) is an emerging respiratory pathogen associated with severe pneumonia and acute respiratory disease, yet no licensed vaccine is currently available. Here, we evaluated the immunogenicity and protective efficacy of an inactivated HAdV-55 vaccine formulated with aluminum hydroxide (iHAdV-55 + AH) in cynomolgus macaques. Animals received a prime–boost intramuscular immunization, and HAdV-55-specific binding IgG and neutralizing antibody responses were monitored longitudinally for up to 52 weeks. Protective efficacy was assessed in two independent respiratory challenge studies using combined intranasal and intratracheal inoculation with either a high dose (1 × 10^11^ plaque forming unit: PFU) or a low dose (1 × 10^9^ PFU) of HAdV-55. Vaccination induced HAdV-55-specific binding and neutralizing antibody responses that remained detectable throughout the 52-week study period. Following the high-dose challenge, vaccinated macaques showed reduced pulmonary lesion burden on chest computed tomography, lower relative lung weights, and attenuated histopathological changes compared with phosphate-buffered saline (PBS) controls. In the low-dose challenge model, where overall disease manifestations were milder, vaccinated animals consistently exhibited fewer radiologic abnormalities, reduced inflammatory cell infiltration, and decreased viral shedding in nasal swabs. No evidence of vaccine-enhanced respiratory disease was observed in either challenge setting. Together, these findings indicate that iHAdV-55 + AH is immunogenic and attenuates HAdV-55-induced pulmonary pathology in this non-human primate model, supporting further development of inactivated vaccine strategies against severe HAdV-55 infection.

## Highlights

• Inactivated HAdV-55 vaccine with alum adjuvant induced durable neutralizing antibodies for 52 weeks in cynomolgus macaques.
• Vaccination significantly attenuated pulmonary lesion burden after high-dose (10¹¹ PFU) HAdV-55 respiratory challenge.
• Protective efficacy was confirmed in a low-dose (10⁹ PFU) model with reduced CT abnormalities and viral shedding.
• Higher pre-challenge neutralizing antibody titers correlated inversely with radiologic and histopathologic disease severity.
• No evidence of vaccine-associated enhanced respiratory disease was observed in either challenge setting.

## 1 Introduction

Human adenoviruses (HAdVs) are non-enveloped, double-stranded DNA viruses belonging to the family Adenoviridae and are currently classified into seven human-associated Mastadenovirus species according to the latest ICTV taxonomy (Lynch and Kajon, 2021; Lion, 2014). HAdVs exhibit broad tissue tropism and cause diverse clinical manifestations, including respiratory tract infections, gastroenteritis, conjunctivitis, and less commonly meningoencephalitis. Mastadenovirus betae, Mastadenovirus cestri, and Mastadenovirus epsi are primarily associated with respiratory disease, whereas Mastadenovirus foxi and Mastadenovirus gami are mainly associated with acute gastroenteritis and diarrhea. Although infections are often self-limiting in immunocompetent individuals across all ages, severe disease can develop in vulnerable groups such as young children, immunocompromised patients, and elderly in long-term care facilities. Critically, outbreaks in crowded settings like military recruit training camps have led to severe pneumonia and fatalities, highlighting the ongoing public health burden of pathogenic respiratory HAdVs (Kim et al., 2024).

HAdV-55 is an emerging, highly virulent respiratory pathogen arising from hexon gene recombination between HAdV-11 and HAdV-14 (Walsh et al., 2010). Initially isolated in Spain in 1969, HAdV-55 has evolved from sporadic cases to a major cause of acute respiratory disease (ARD) and severe pneumonia worldwide (Hierholzer et al., 1974; Ko et al., 2021; Yoo et al., 2017; Salama et al., 2016; Xu et al., 2018; Zhang et al., 2012; Lafolie et al., 2016; Kajon et al., 2010; Lu et al., 2020; Cao et al., 2014). Outbreaks are common in schools and military camps, with >538 documented cases in South Korean military facilities alone (2013–2018). Its enhanced pathogenicity derives from tropism for airway and alveolar stem cells, plus high-affinity fiber protein binding to cellular receptors, resulting in more severe symptoms than other genotypes (Lafolie et al., 2016; Cao et al., 2014; Zhang et al., 2024; Zhao et al., 2023). Given its pandemic potential and fatalities even in immunocompetent hosts, HAdV-55 represents a significant threat akin to other major respiratory pathogens—yet no licensed vaccine exists (Stasiak and Stehle, 2020; Wu et al., 2002; Sumida et al., 2005; Tian et al., 2011).

Development of HAdV-55 vaccine has largely focused on hexon-directed strategies, including vector-based approaches. Studies using recombinant HAdV-3 vectors engineered to express HAdV-55 hexon epitopes have demonstrated the induction of HAdV-55–specific nAb responses in mice, supporting the immunogenic potential of hexon-targeted vaccines (Liu et al., 2018; Tian et al., 2018). In addition, analyzes of human sera with neutralizing activity against HAdV-55 revealed antibody responses directed against both hexon and fiber proteins, suggesting that vaccine strategies incorporating multiple capsid antigens may provide broader protection against HAdV-55 infection (Feng et al., 2018). Consequently, whole-virus platforms have been pursued. These include replication-incompetent vectors generating type-specific nAb responses in mice and macaques, and aluminum hydroxide (AH)-formulated inactivated HAdV-55 (iHAdV-55) that elicits neutralizing antibody responses in a murine model (Feng et al., 2026; Seo et al., 2024). Despite these promising immunogenicity data, the protective efficacy of HAdV-55 vaccine candidates has not been fully evaluated due to the lack of an animal infection model that recapitulates key features of human disease. To address this limitation, we recently established a cynomolgus macaque model of HAdV-55 infection, in which infected animals developed characteristic pulmonary pathology, including peri‑bronchial consolidation and ground-glass opacities on computed tomography, as well as inflammatory lesions on histopathological analysis, thereby providing a relevant platform for evaluating vaccine-induced protection (Seo et al., 2026).

Here, we evaluated the immunogenicity and protective efficacy of iHAdV-55 + AH in cynomolgus macaques. Vaccination induced HAdV-55–specific neutralizing antibody responses that remained detectable for up to 52 weeks and reduced pulmonary pathology following both high-dose (1 × 10¹¹ plaque-forming unit (PFU)) and low-dose (1 × 10⁹ PFU) HAdV-55 challenge. Importantly, no evidence of vaccine-enhanced disease was observed. Together, these findings support iHAdV-55 + AH as a candidate warranting further evaluation against severe HAdV-55 respiratory disease.

## 2 Methods

### 2.1 Cells

Vero (CLB-VERO01WCB-073; MFDS, Korea) and A549 (KCLB 10,185; KCLB, Korea) cells were maintained in minimun essential medium (MEM) supplemented with 10% heat-inactivated fetal bovine serum (FBS) and 1% penicillin–streptomycin at 37 °C in 5% CO₂ and passaged every 3–4 days. Cell identity and sterility/mycoplasma status were verified before use.

### 2.2 Virus culture, purification, and titration

HAdV-55 was isolated from respiratory clinical specimens as described previously. (Seo et al., 2024) A549 cells were infected at multiplicity of infection (MOI) 2.5 in Opti-MEM. At 2–4 days post-infection (dpi), supernatants were harvested, clarified (3000 rpm, 10 min, 4 °C), filtered (0.45 μm), and stored at −80 °C. The stored virus stocks were amplified as previously reported (Seo et al., 2024). Virus was purified by flow-through chromatography using Capto Core 700 and concentrated by ultrafiltration. Infectious titers were determined by plaque assay on A549 cells: monolayers were inoculated with serially diluted virus (2 h), overlaid with MEM/2% FBS/0.8% agarose, incubated 7 days, fixed, stained, and reported as PFU/mL using the formula: PFU = [(plaques/well) × dilution factor] / inoculum volume.

### 2.3 Preparation of vaccine candidate

For vaccine bulk, Vero cells in 10-layer CellSTACKs were infected with HAdV-55 (MOI 5) and incubated for 5 days. Harvests underwent two freeze–thaw cycles (−70 °C), clarification (4750 rpm, 20 min, 4 °C), and sterile filtration through a 0.2 μm Sartopore® 2 MidiCap filter, with integrity testing before and after filtration. Filtrates were collected in sterile Flexboy® bags and stored at −70 °C until purification by Capto Core 700 flow-through chromatography. Virus-containing fractions were pooled, and inactivation was performed by adding formaldehyde (final 0.1 M) and incubating at 37 °C for 24 h with mixing, followed by ultrafiltration/diafiltration into PBS using a 300 kDa membrane (15 × diafiltration). The successful inactivation of the virus was confirmed by plaque assay as described above in the virus titration method. The active pharmaceutical ingredient was adjusted to 1000 internal unit (IU)/mL (equivalent to 4 × 10⁷ PFU of live virus as determined by hexon enzyme-linked immunosorbent assay (ELISA)). Aluminum hydroxide gel was added to 0.5 mg/mL (5% v/v), mixed to homogeneity, aseptically filled (1.2 mL/vial; 1.160–1.240 mL), stoppered, sealed, and stored at 2–8 °C.

### 2.4 Animal care, housing conditions, and ethics statement

Fourteen male cynomolgus macaques (Macaca fascicularis; 3–4 years) were housed individually in a BSL-2 facility under controlled temperature/humidity and a 12-h light/dark cycle, with standard diet and water ad libitum. Studies were conducted at an AAALAC-accredited facility and approved by the Keyprime Research IACUC (IACUC-AP-23–023–1 and IACUC-AP-25–024). All infectious work was performed in BSL-2 and waste was autoclaved.

### 2.5 Study design

Baseline screening confirmed seronegativity for HAdV-55 nAb. Experiment 1 (immunogenicity/durability) used two-dose intramuscular vaccination (day 0 and 28) with 125 or 250 IU (n = 2/group) and followed animals for 52 weeks with clinical observations, hematology, and serology. Experiment 2 (challenge) used two-dose intramuscular vaccination with iHAdV-55 (250 IU) + AH (n = 3), followed by HAdV-55 challenge under ketamine anesthesia (5 mg/kg, IM): high dose 1 × 10¹¹ PFU or low dose 1 × 10⁹ PFU delivered intranasally (0.5 mL/nostril) plus intratracheally (4 mL). Animals were monitored for 14 dpi and lungs were collected at 14 dpi for pathology.

### 2.6 Clinical monitoring and sampling

Animals were assessed at least twice daily. Body weight was recorded daily and food intake weekly. A blinded, predefined clinical scoring system captured general appearance, respiratory status, activity, and other parameters (reported as mean ± SD). Nasal and oral swabs were collected at 0, 1, 2, 4, 6, 8, 10, 12, and 14 dpi into transport medium and stored at −70 °C. Respiratory rate, rectal temperature, SpO₂, and heart rate were measured using standard veterinary procedures.

### 2.7 Chest computed tomography (CT) and quantitative analysis

CT was performed under ketamine/isoflurane anaesthesia in the supine position during free breathing (0.6-mm slice thickness; 120 kV; 100 mA; pitch 1.4; B50f medium sharp reconstruction kernel). Lung segmentation and attenuation-based volumetric analysis were performed using Siemens Inveon Research Workplace (IRW, 4.2 version, Siemens Medical Solution, USA, Inc.). Both lungs were segmented as anatomical structures bounded by the visceral pleura, including aerated parenchyma, pulmonary vessels, airway walls, and non-aerated regions such as consolidation and atelectasis; the trachea and main bronchi were excluded. Initial masks were generated by thresholding of aerated parenchyma, followed by morphological closing and slice-by-slice manual correction to incorporate pulmonary vessels, airway walls and pleura-adjacent consolidation into the mask. Total lung volume (TLV) was computed as the voxel volume of this mask (nominal window, −1024 to +150 Hounsfield unit (HU)). Within the same mask, voxels in the nominal −300 to +150 HU window were quantified as the high-attenuation volume (HAV), following thresholds applied to quantitative assessment of consolidative pulmonary opacity (Du et al., 2020). HAV is reported both in absolute terms (mm³) and as a fraction of TLV (%HAV). Importantly, HAV is not zero in normal lungs: this window necessarily captures pulmonary vessels, airway walls, hilar and perihilar structures, gravity-dependent atelectasis that develops under general anaesthesia, and partial-volume voxels at tissue–air interfaces. Baseline HAV therefore represents anatomical and anaesthesia-related background rather than pathological lesion; the reference range measured in all unchallenged animals in this study is given in Supplementary Table S1 and illustrated in Supplementary Figure S1. Vaccine effects were accordingly evaluated as change from each animal’s own pre-challenge scan, expressed as the absolute change (ΔHAV = HAV at 14 dpi − HAV at −1 dpi) and as the percentage change in high-attenuation lung volume (ΔHAV%), calculated as [(post − pre)/pre] × 100. All pre-challenge scans were additionally reviewed for the presence of consolidation or ground-glass opacity. Because of the small group sizes, CT metrics are reported descriptively, with individual animal values shown throughout, and were not subjected to hypothesis testing.

### 2.8 Histology and immunohistochemistry

Whole lung lobes were fixed in 10% neutral buffered formalin, paraffin embedded, sectioned (4 μm), and H&E stained. Blinded pathology scores (0–5) reflected inflammatory infiltration and tissue damage. For IHC, deparaffinized sections were stained using an horseradish peroxidase (HRP)/3,3'-diaminobenzidine system with antibodies to CD3 (clone CD3–12), CD20 (polyclonal), CD68 (clone KP1), and Ki-67 (clone B56). Whole-slide images were acquired and positive area (%) was quantified in ImageJ from five representative regions per animal.

### 2.9 Viral load quantification

Nasal and oral swab samples were collected on days 0, 1, 2, 4, 6, 8, 10, 12, and 14 dpi for evaluation of viral load and plaque formation. Each swab was placed in virus transport medium (CTM; Noble Biosciences Inc., Hwaseong-si, Korea) and stored at −70 °C until further analysis. Viral genomes in swabs and blood were quantified by real-time quantitative polymerase chain reaction (RT-PCR) after extraction (Viral Gene-spin kit) using TB Green chemistry with primers 5′-CAGTGGATTGCAGAAGGT-3′ and 5′-CAGCATAAATCGGTTTACTTTCTTC-3′ under 95 °C/57 °C cycling (40 cycles); copy numbers were interpolated from a standard curve. Infectious virus in swabs was quantified by plaque assay on A549 cells as described above.

### 2.10 Serology

Binding IgG was measured by ELISA using purified HAdV-55–coated plates and an HRP-conjugated anti-monkey IgG detection antibody; this assay detects IgG only and does not capture IgM. Endpoint titers were the highest dilution with OD₄₅₀ ≥ 0.2 above background and are reported as log₂. Vaccine potency (IU) was measured by hexon antigen ELISA; for iHAdV-55 + AH, alum was desorbed in extraction buffer (0.4 M sodium phosphate dibasic, 3 mM EDTA, 0.1% Tween-20, 0.1% gelatin; pH 8.5) at 37 °C for 3 h, followed by centrifugation. Neutralization was measured by plaque reduction neutralization test (PRNT) as below.

### 2.11 Plaque reduction neutralization test (PRNT)

Neutralizing antibodies against HAdV-55 were measured by plaque reduction neutralization test (PRNT) (Seo et al., 2026). Briefly, serially diluted sera were incubated with 100 PFU of HAdV-55 for 60 min at 37 °C and added to A549 cell monolayers for 2 h. Cells were then overlaid with MEM containing 2% FBS and 0.8% agarose and incubated for 7 days. After fixation and crystal violet staining, neutralizing titers were calculated as PRNT₅₀ values, defined as the reciprocal serum dilution that reduced plaque numbers by 50% relative to virus-only controls.

### 2.12 Statistics

All analyses were performed using GraphPad Prism v10.4.0 (GraphPad Software). Data are presented as mean ± SEM. Given the small number of animals per group (n = 2–3), quantitative endpoints are reported descriptively with individual animal values displayed; formal significance testing was not applied to the immunohistochemical morphometry, for which multiple image fields per animal precluded valid animal-level inference.

## 3 Results

### 3.1 Vaccine-induced binding and neutralizing antibody responses in cynomolgus macaques

**Fig. 1:**
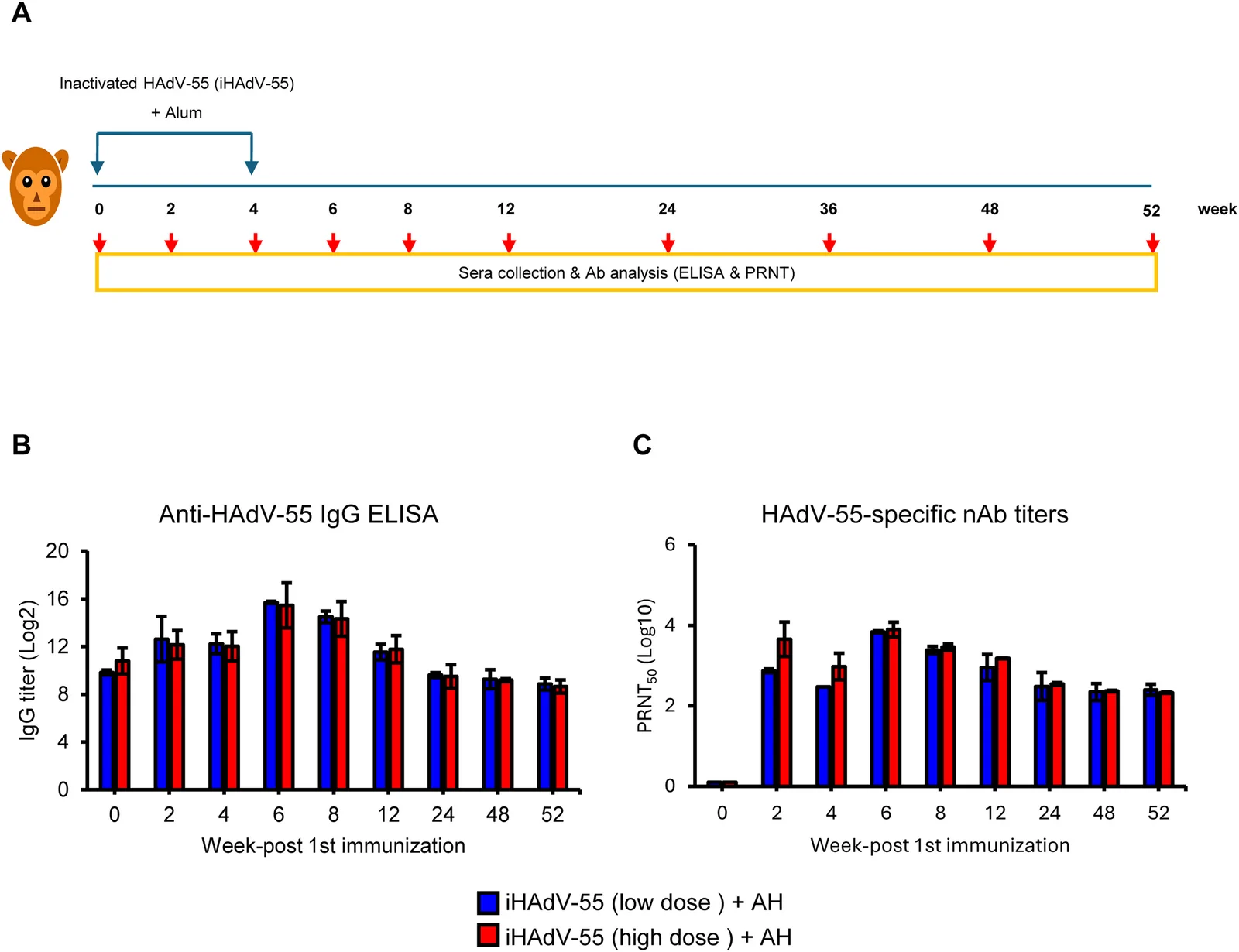
Immunization schedule and durability of humoral immunity induced by an inactivated HAdV-55 vaccine in cynomolgus macaques. (A) Study design. Cynomolgus macaques were immunized twice (weeks 0 and 4) with inactivated HAdV-55 (iHAdV-55) formulated with aluminum hydroxide (AH) at either a low (125 IU) or high dose (250 IU); sera were collected at the indicated time points for antibody analyzes. (B) Anti-HAdV-55 IgG titers measured by ELISA and expressed as log2 titers. (C) HAdV-55-specific neutralizing antibody (nAb) titers measured by plaque reduction neutralization test (PRNT) and expressed as log10 titers. Bars indicate group means with error bars showing dispersion as plotted in the figure. n = 2 per group.

Prior to evaluating the protective efficacy of inactivated HAdV-55 formulated with aluminum hydroxide (iHAdV-55 + AH) in cynomolgus macaques, we first assessed the immunogenicity of the vaccine candidate by measuring HAdV-55–reactive binding antibody (bAb) and neutralizing antibody (nAb) responses. Accordingly, macaques were immunized intramuscularly with either a high dose (250 IU, n = 2) or low dose (125 IU, n = 2) of iHAdV-55 + AH in a prime–boost regimen with a 4-week interval, and Ab titers were analyzed by ELISA and plaque reduction neutralization test (PRNT) (Fig. 1A). Prior to immunization, all macaques exhibited detectable levels of HAdV-55–reactive binding antibodies (bAbs) by ELISA (Fig. 1B). Baseline IgG titers remained detectable throughout the longitudinal observation period and showed only moderate increases following vaccination, particularly after the second immunization (weeks 4–8). These baseline ELISA signals may reflect pre-existing cross-reactive Abs induced by prior exposure to other adenoviruses, as serological cross-reactivity between simian and human adenoviruses has previously been reported (Willimzik et al., 1981). In contrast, baseline HAdV-55–specific neutralizing antibody (nAb) titers (PRNT₅₀) were minimal or undetectable prior to vaccination (Fig. 1C), indicating that the pre-existing ELISA-reactive antibodies were largely non-neutralizing against HAdV-55. The dissociation between high baseline binding and undetectable baseline neutralization is consistent with the antigenic architecture of adenoviruses, in which type-specific neutralization is largely conformation-dependent and directed against hexon hypervariable regions and type-specific fiber determinants, whereas cross-reactive, non-neutralizing antibodies recognize conserved genus- and species-level epitopes that are efficiently detected on purified antigen by ELISA but do not neutralize the intact virion (Feng et al., 2018; Willcox and Mautner, 1976). These pre-existing HAdV-55–reactive antibodies are therefore most plausibly directed against conserved, non-hypervariable hexon determinants, although their precise specificity was not experimentally mapped in this study and would require antigen-resolved serology to define. Following the booster immunization, nAb titers peaked at 6–8 weeks and remained measurable throughout the 52-week observation period, with no appreciable differences between the two dose groups. Collectively, these results indicate that iHAdV-55 + AH induces durable HAdV-55–reactive bAb and type-specific nAb responses in cynomolgus macaques at both dose levels.

### 3.2 High-dose challenge study schema and post-challenge humoral antibody responses

**Fig. 2:**
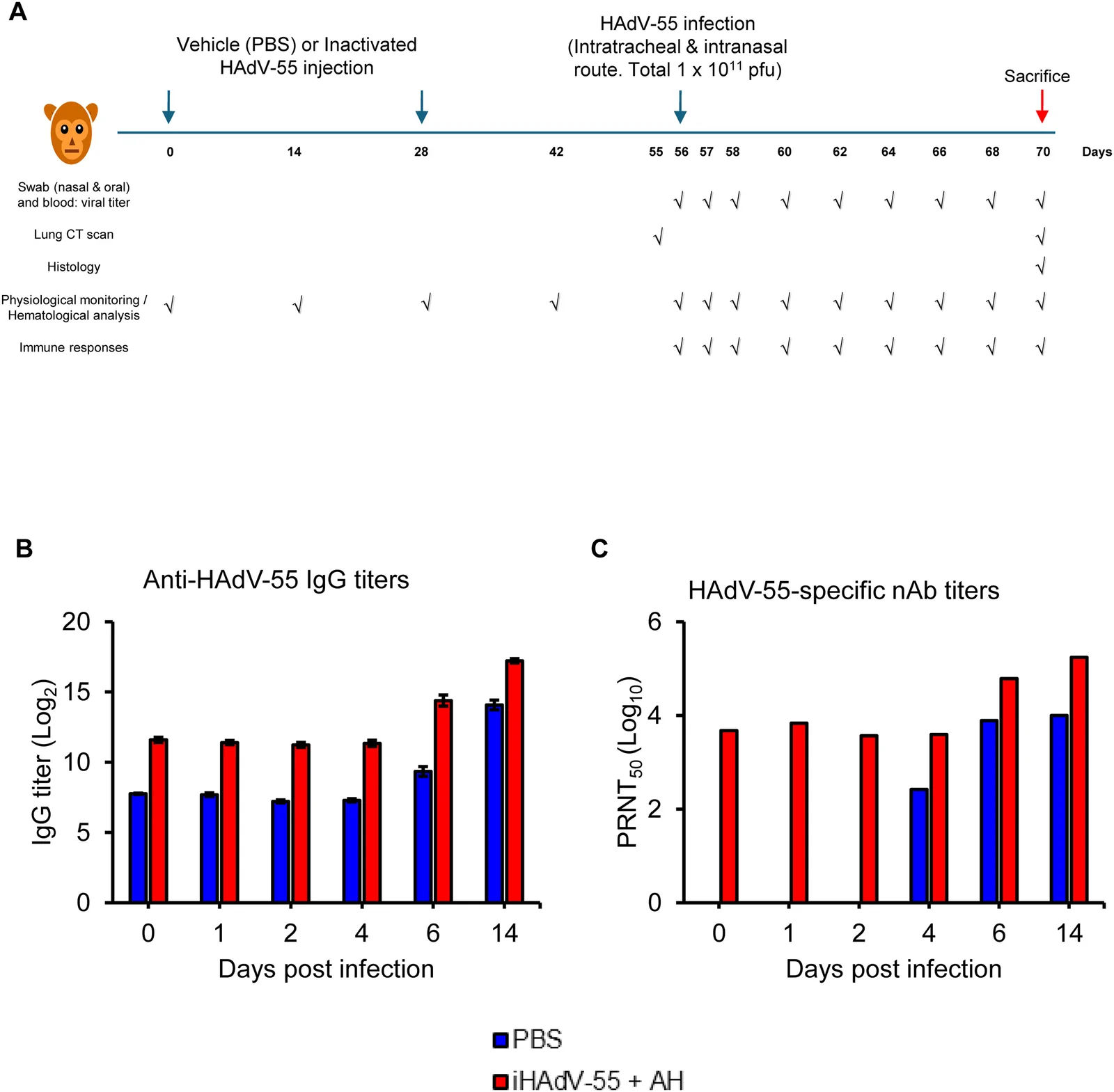
Study scheme for HAdV-55 challenge and longitudinal sampling, and post-challenge humoral responses. (A) Challenge study overview. Macaques received vehicle (PBS, n = 2) or iHAdV-55 + AH (250 IU, n = 3) on days 0 and 28 (days post first immunization), followed by HAdV-55 (1 × 10¹¹ PFU) challenge via intranasal and intratracheal routes on day 56 (defined as 0 days post-infection, dpi); swabs (nasal/oral), blood, physiological/hematological monitoring, and immune measurements were collected at the indicated time points, and animals were sacrificed on day 70 (14 dpi). Study days (post first immunization) and the corresponding dpi are both indicated on the timeline. (B) Anti-HAdV-55 IgG titers (log2) in PBS- versus iHAdV-55 + AH-treated animals measured by ELISA at the indicated days post infection (dpi). (C) HAdV-55–specific nAb titers (PRNT_50_; log10) measured over time post challenge. Bars represent group means with error bars as shown.

To evaluate the protective efficacy of iHAdV-55 + AH, macaques were immunized with either iHAdV-55 + AH (n = 3) or PBS (n = 2) and subsequently challenged with a high dose of HAdV-55 (1 × 10^11^ PFU) on day 56. Post-challenge, animals were monitored via longitudinal clinical assessments and serial sampling (Fig. 2A). ELISA revealed that vaccinated macaques maintained significantly higher HAdV-55-specific binding IgG titers compared to PBS-treated controls. These animals also exhibited an anamnestic response, characterized by rapidly rising antibody levels that peaked at the terminal time point (Fig. 2B). Correspondingly, nAb titers in the iHAdV-55 + AH group consistently surpassed those of the PBS group at all post-challenge intervals, reaching maximal levels by the end of the study. In contrast, the PBS group showed delayed and lower nAb induction (Fig. 2C). Of note, the binding IgG ELISA detects IgG only; the low-level neutralizing activity detectable in PBS animals at 4 dpi, in the absence of a concurrent rise in binding IgG, is most consistent with an early IgM contribution to neutralization that precedes the class-switched IgG response and is not captured by the IgG-specific ELISA (Unzu et al., 2014). Together, these results demonstrate that immunization of iHAdV-55 + AH not only sustained elevated levels of specific Abs but also primed the immune system for a rapid anamnestic boost upon viral exposure, leading to peak Ab levels.

### 3.3 Clinical course and viral kinetics following high-dose HAdV-55 challenge

**Fig. 3:**
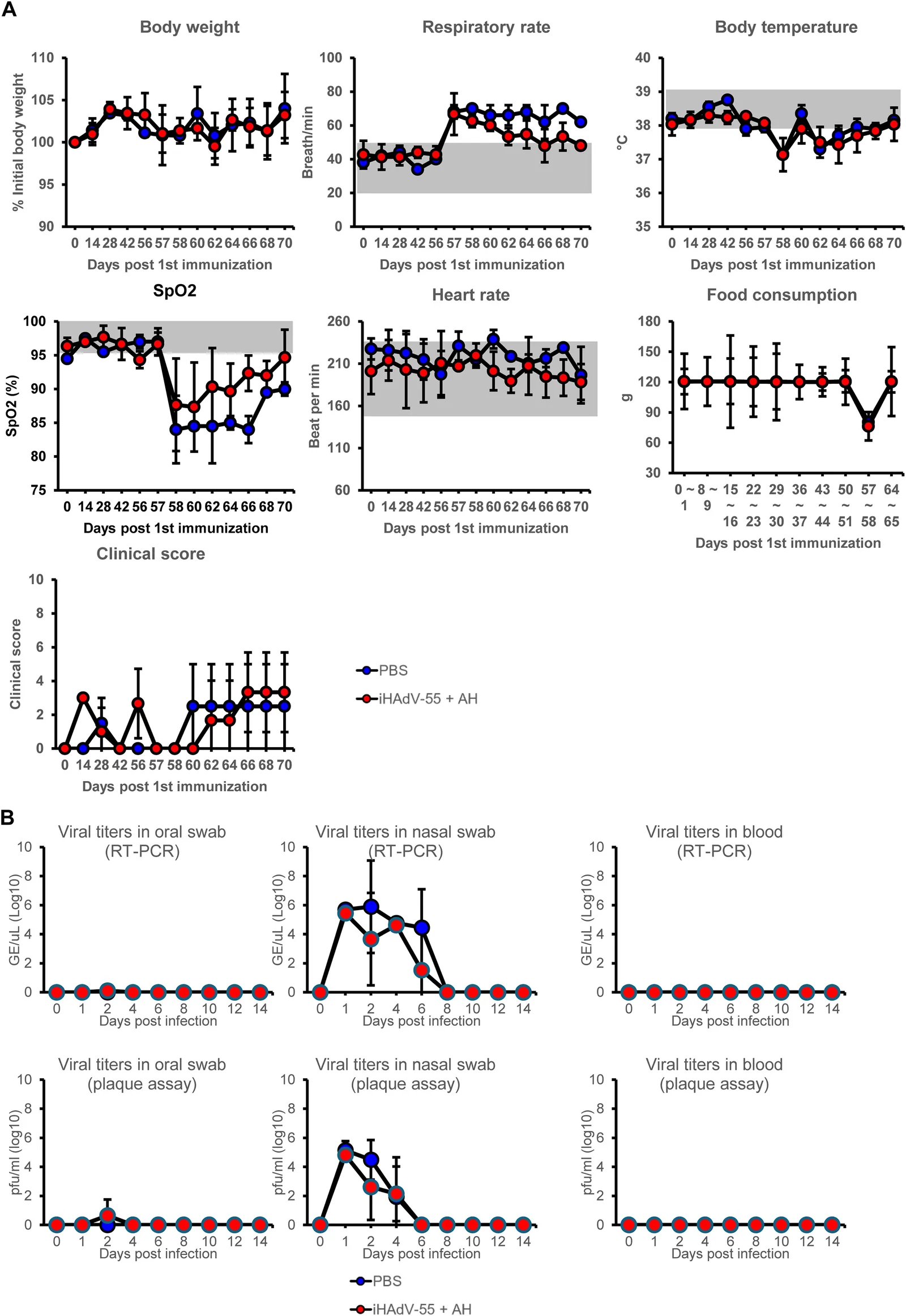
Physiological changes and viral kinetics following HAdV-55 challenge. (A) Longitudinal monitoring of body weight (percent of baseline), respiratory rate, body temperature, peripheral oxygen saturation (SpO₂), heart rate, food consumption, and composite clinical score in PBS- and iHAdV-55 + AH-treated macaques; the x-axis is expressed as days post first immunization, with challenge performed on day 56. Shaded regions indicate reference/normal ranges as displayed. (B) Viral titers in oral swab, nasal swab, and blood samples measured by RT-PCR (reported as genome equivalents; log₁₀) and by plaque assay (reported as PFU/mL; log₁₀); the x-axis is expressed as days post-infection (dpi), where challenge = 0 dpi (corresponding to day 56 post first immunization) and denotes the pre-challenge baseline sampling time point. Lines/points depict group means with error bars as shown.

Following the challenge with HAdV-55 (1 × 10^11^ PFU), the macaques were monitored for physiological and virological parameters through longitudinal analysis (Fig. 3). Both PBS- and iHAdV-55 + AH-treated animals exhibited increased respiratory rates after HAdV-55 infection (Fig. 3A); however, vaccinated macaques returned to normal ranges more rapidly than PBS controls. Similarly, saturation pulse oxygen (SpO₂) declined in both groups following HAdV-55 infection but recovered more quickly in the iHAdV-55 + AH immunized group than in the PBS-treated group. Other physiological parameters—including body weight, temperature, heart rate, food consumption, and clinical scores—remained comparable between groups. Virological analysis indicated that HAdV-55 was localized mainly to nasal swabs, with viral loads and shedding kinetics in the iHAdV-55 + AH group mirroring those of the PBS group (Fig. 3B). Oral shedding was minimal and no significant viremia was detected in either group. These results indicate that while iHAdV-55 + AH vaccination does not markedly suppress viral replication or shedding, it provides a functional benefit by facilitating faster physiological recovery following HAdV-55 infection.

### 3.4 Vaccination attenuates pulmonary and histopathological severity after high-dose HAdV-55 challenge

**Fig. 4:**
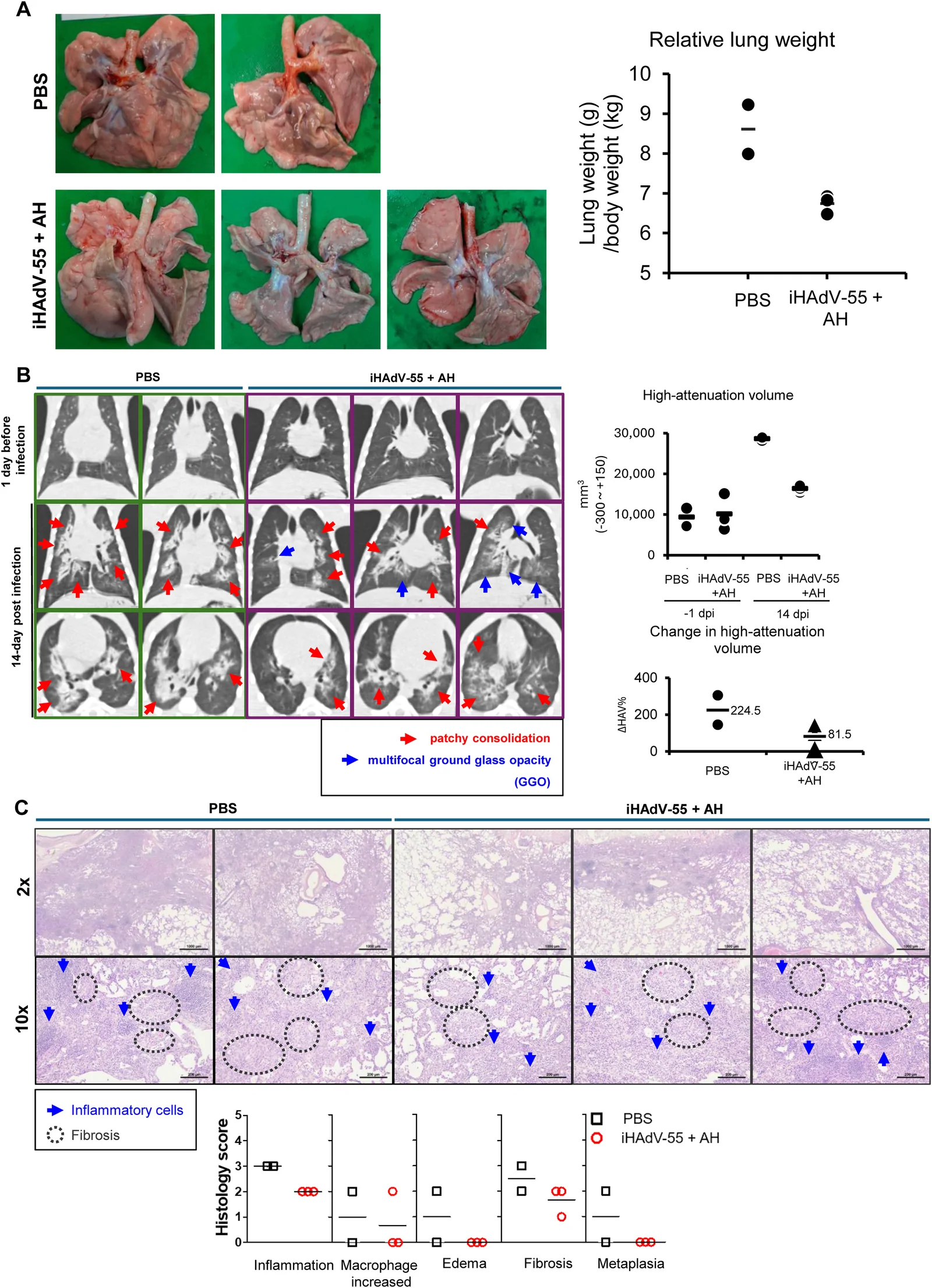
Vaccine-associated reduction of pulmonary pathology after HAdV-55 challenge. (A) Gross lung appearance at necropsy (representative images by group) and relative lung weight (lung weight normalized to body weight; each symbol represents an individual animal; horizontal line indicates the group mean). (B) Representative chest CT images collected 1 day before challenge and at 14 dpi in PBS and iHAdV-55 + AH groups; arrows indicate radiologic abnormalities, including patchy consolidation (red) and multifocal ground-glass opacity (GGO; blue). Quantification of the high-attenuation volume at −1 dpi and 14 dpi is presented, with the corresponding percentage change in high-attenuation volume (ΔHAV%) shown in the right panel. (C) Representative H&E-stained lung sections (2 × and 10 ×) demonstrating inflammatory cell infiltration (blue arrows) and fibrosis (dashed outlines), with semi-quantitative histopathology scores for inflammation, increased macrophages, edema, fibrosis, and metaplasia; symbols denote individual animals and horizontal lines indicate group means. HAV: high attenuation volume.

To assess the protective efficacy of the vaccine against HAdV-55-induced lung disease, we performed comprehensive analyzes of lung weight, chest computed tomography (CT), and histopathology (Fig. 4). At 14 days post-infection (dpi), vaccinated macaques (n = 3) exhibited lower relative lung weights compared to the PBS-treated control group (n = 2) (Fig. 4A). Given that increased lung weight following viral infection is typically a surrogate marker for inflammation-associated edema and cellular infiltration (Feng et al., 2020), these findings suggest that iHAdV-55 + AH immunization effectively prevents severe pulmonary inflammation.

Consistent with these findings, longitudinal chest CT imaging, conducted at baseline and 14 dpi, revealed marked pneumonia-associated abnormalities in the PBS control group, characterized by patchy consolidation and multifocal ground-glass opacities (GGO) (Fig. 4B). In contrast, vaccinated animals showed a marked reduction in radiologic lesion burden. Quantitative CT analysis was performed as change from each animal’s own pre-challenge scan. At 1 day before challenge, HAV was comparable between groups (PBS, 9338 mm³, 7.6% of TLV; iHAdV-55 + AH, 10,103 mm³, 5.9% of TLV). These values are consistent with the anatomical background of this HU compartment measured across all 10 unchallenged animals in the two challenge studies (median 5.3% of TLV, interquartile range 4.8–6.2%, range 4.2–9.5%; mean ± SD, 9427 ± 2619 mm³; Supplementary Table S1), and no consolidation or ground-glass opacity was identified on review of any pre-challenge scan (Fig. 4B, upper row; Supplementary Fig. S1). By 14 dpi, HAV in PBS-treated animals had increased approximately threefold (28,566 mm³; 19.0% of TLV), whereas the increase in vaccinated animals was substantially smaller (16,329 mm³; 8.9% of TLV). The corresponding percentage change in high-attenuation lung volume (ΔHAV%) was 224.5 in PBS controls versus 81.5 in vaccinated animals. Individual animal values are provided in Supplementary Table S2a. Given the small group sizes (PBS, n = 2; iHAdV-55 + AH, n = 3), individual animal values are plotted in Fig. 4B so that readers can assess the variability underlying these group-level differences; these differences should therefore be interpreted as small-sample estimates rather than as precisely quantified effects.

Histopathological evaluation confirmed the radiologic findings, providing cellular-level evidence of protection (Fig. 4C). Lung sections from PBS-treated controls displayed extensive inflammatory injury, including dense inflammatory cell infiltration and early signs of fibrotic changes. Conversely, lung tissues from vaccinated animals exhibited attenuated pathology with preserved alveolar structures. Semi-quantitative scoring across multiple pathological domains consistently demonstrated lower severity in the vaccinated group, reinforcing the vaccine's role in mitigating virus-induced tissue damage. Collectively, these data indicate that iHAdV-55 + AH vaccination confers significant protection against HAdV-55-induced pulmonary disease.

### 3.5 Immunohistochemical quantification following high-dose HAdV-55 challenge

**Fig. 5:**
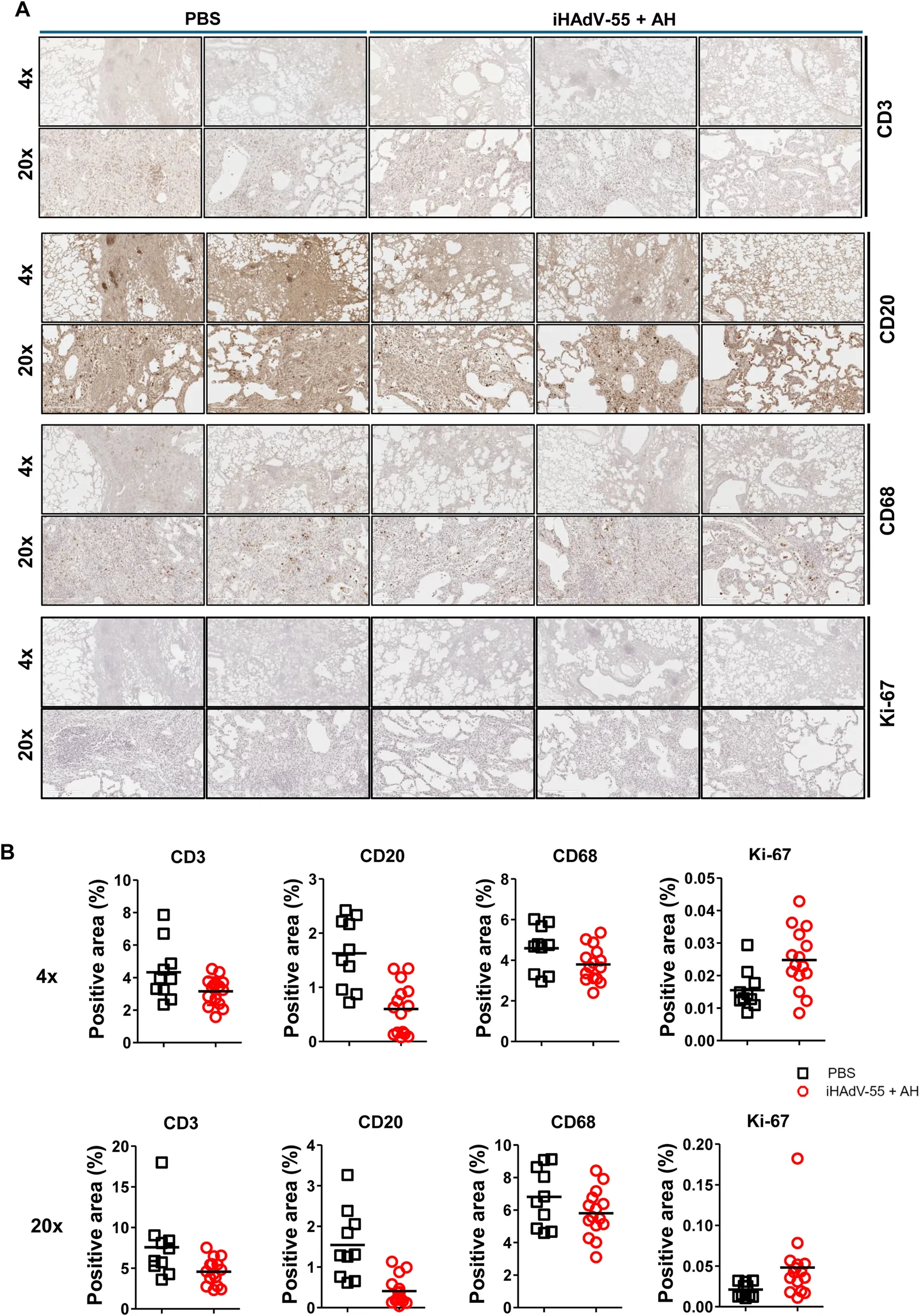
Lung immunohistochemistry after high-dose HAdV-55 challenge. (A) Representative lung sections from PBS-treated controls and iHAdV-55 + AH–immunized animals stained for CD3 (T cells), CD20 (B cells), CD68 (macrophages), and Ki-67 (proliferating cells), shown at 4 × and 20 × magnification. (B) Quantification of marker-positive area (positive area, %) at 4 × and 20 ×. Each symbol represents an individual image field (PBS, black squares; iHAdV-55+AH, red circles), with horizontal bars indicating group means. Due to the small sample size per group (n=2−−3), formal statistical testing was not performed, and results are described qualitatively.

To characterize lung immune-cell infiltration and proliferative activity following high-dose challenge, we performed IHC staining for CD3, CD20, CD68, and Ki-67 with quantitative image analysis (Fig. 5A). The immunohistochemical analysis demonstrated marked lymphoid and myeloid cell infiltration in the lungs of PBS-treated animals, whereas immune cell accumulation was substantially reduced in vaccinated animals. At both 4 × and 20 × magnification, PBS-challenged lungs showed dense CD3⁺ T-cell and CD20⁺ B-cell aggregates around bronchi and within perivascular and interstitial regions, while these infiltrates were less prominent and more scattered in the iHAdV-55 + AH group. Consistently, morphometric quantification revealed a relatively lower CD20⁺ positive area in vaccinated lungs compared with PBS controls at both magnifications, with a more modest reduction in CD3⁺ areas, indicating that vaccination more effectively limited B-cell–rich inflammatory foci (Fig. 5B). CD68⁺ macrophages were abundantly present in both groups, and their positive area did not differ appreciably, suggesting that resident or recruited macrophage populations were less influenced by prior immunization. The proportion of Ki-67⁺ proliferating cells was higher in lungs from iHAdV-55 + AH–immunized animals than in PBS controls at both 4 × and 20 × magnification. Because Ki-67 is a pan-proliferation marker, the cellular identity of these proliferating cells could not be determined from this staining alone, and the finding is reported here without inference as to its origin.

### 3.6 Humoral responses following low-dose HAdV-55 challenge

Although iHAdV-55 + AH immunization effectively mitigated pulmonary pathology following high-dose challenge, certain physiological parameters and nasal viral titers did not significantly differ from those in PBS-treated controls. We hypothesized that the initial challenge dose of 1 × 10^11^ PFU might have been excessively high, potentially masking the protective efficacy of the vaccine in terms of systemic physiological changes and virological clearance. To address this and more accurately evaluate the vaccine’s protective potential under different infectious burdens, we further investigated its efficacy using a relatively lower challenge dose of 1 × 10^9^ PFU of HAdV-55.

**Fig. 6:**
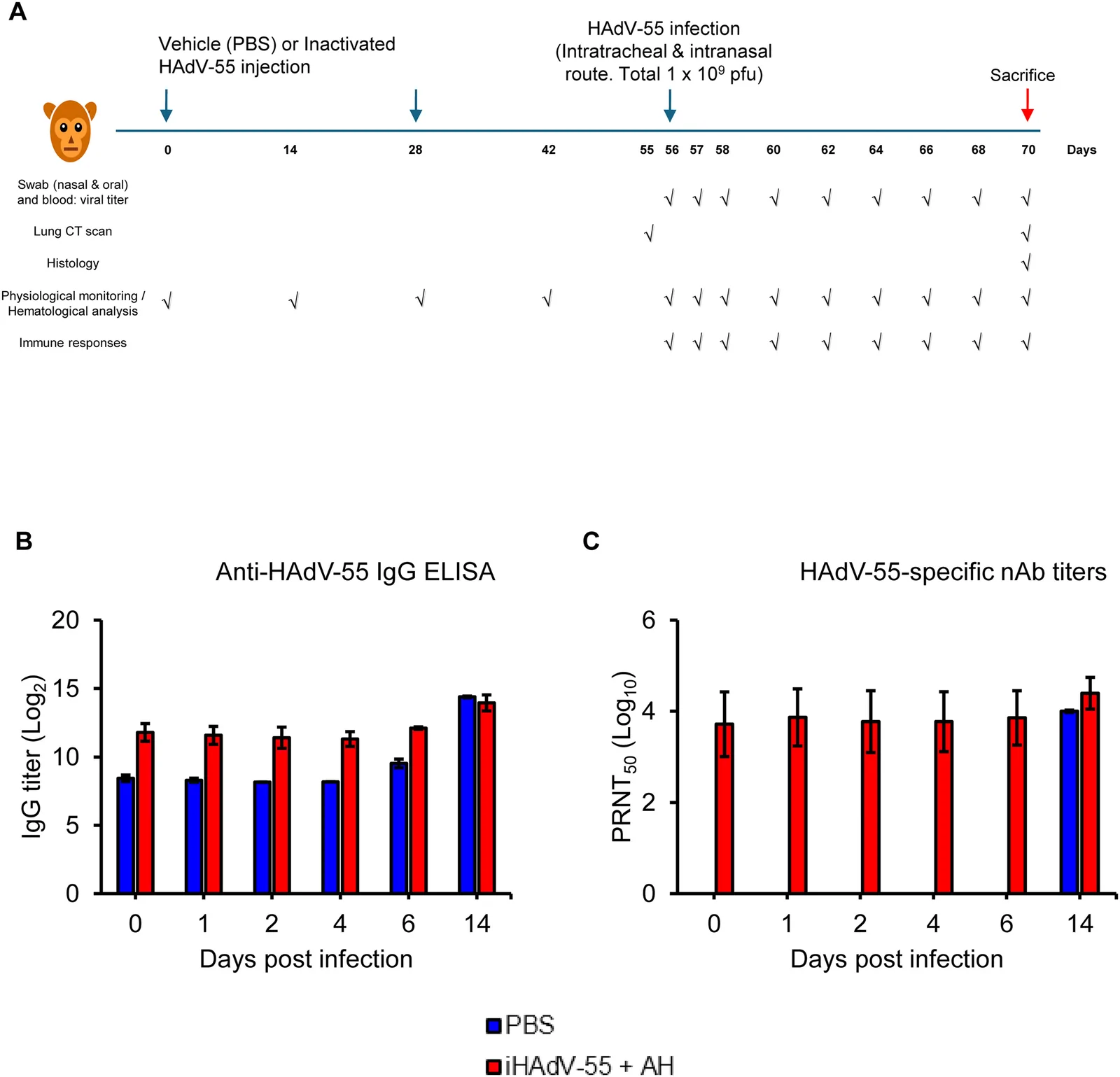
Humoral responses following low dose HAdV-55 (1 × 10^9^ PFU) challenge in PBS versus iHAdV-55 + AH groups. (A) Experimental scheme for the low-dose HAdV-55 (1 × 10⁹ PFU) challenge study. Macaques were immunized with either vehicle (PBS, n = 2) or iHAdV-55+AH (250 IU, n = 3) on days 0 and 28 (days post first immunization). On day 56 (defined as 0 days post-infection, dpi), animals were challenged with 1 × 10⁹ PFU of HAdV-55 via combined intranasal and intratracheal routes. Study days (post first immunization) and the corresponding dpi are both indicated on the timeline. (B) Anti-HAdV-55 IgG titers (ELISA; log_2_) measured at the indicated dpi. (C) HAdV-55–specific nAb titers (PRNT_50_; log_10_) measured at the indicated dpi. Bars represent group means with error bars as shown.

In the low-dose challenge study (1 × 10^9^ PFU), HAdV-55–reactive bAb and HAdV-55-specific nAb responses were monitored longitudinally from 0 to 14 dpi (Fig. 6A). At 0 dpi, macaques in the iHAdV-55 + AH group (n = 3) exhibited higher anti–HAdV-55 IgG ELISA titers than PBS-treated controls (n = 2), and these elevated binding titers were maintained through the early post-challenge period (1–6 dpi), followed by a further increase at 14 dpi (Fig. 6B). By contrast, PBS-treated animals showed lower IgG titers during 0–6 dpi, with a marked rise by 14 dpi, consistent with de novo induction of bAbs following infection.

HAdV-55-specific nAb was detectable at baseline in vaccinated animals and remained stable through 0–6 dpi, with a modest increase by 14 dpi (Fig. 6C). In PBS controls, the nAb titers were undetectable during the early phase (0–6 dpi), with measurable titers only appearing at 14 dpi, indicating delayed nAb induction relative to vaccinated macaques. Within the low-dose experiment, the qualitative patterns of Ab kinetics resembled those observed in the high-dose experiment—namely, elevated and sustained titers in vaccinated animals relative to their concurrent PBS controls. Because the two challenges were performed as independent experiments in separate animal cohorts, we do not compare the magnitude of responses numerically across the two studies; each is interpreted only against its own contemporaneous controls. Collectively, these findings suggest that iHAdV-55 + AH vaccination induces sustained humoral immune responses that persist through the challenge period under both high- and low-dose HAdV-55 infection conditions.

### 3.7 Clinical parameters and viral shedding after low-dose HAdV-55 challenge

**Fig. 7:**
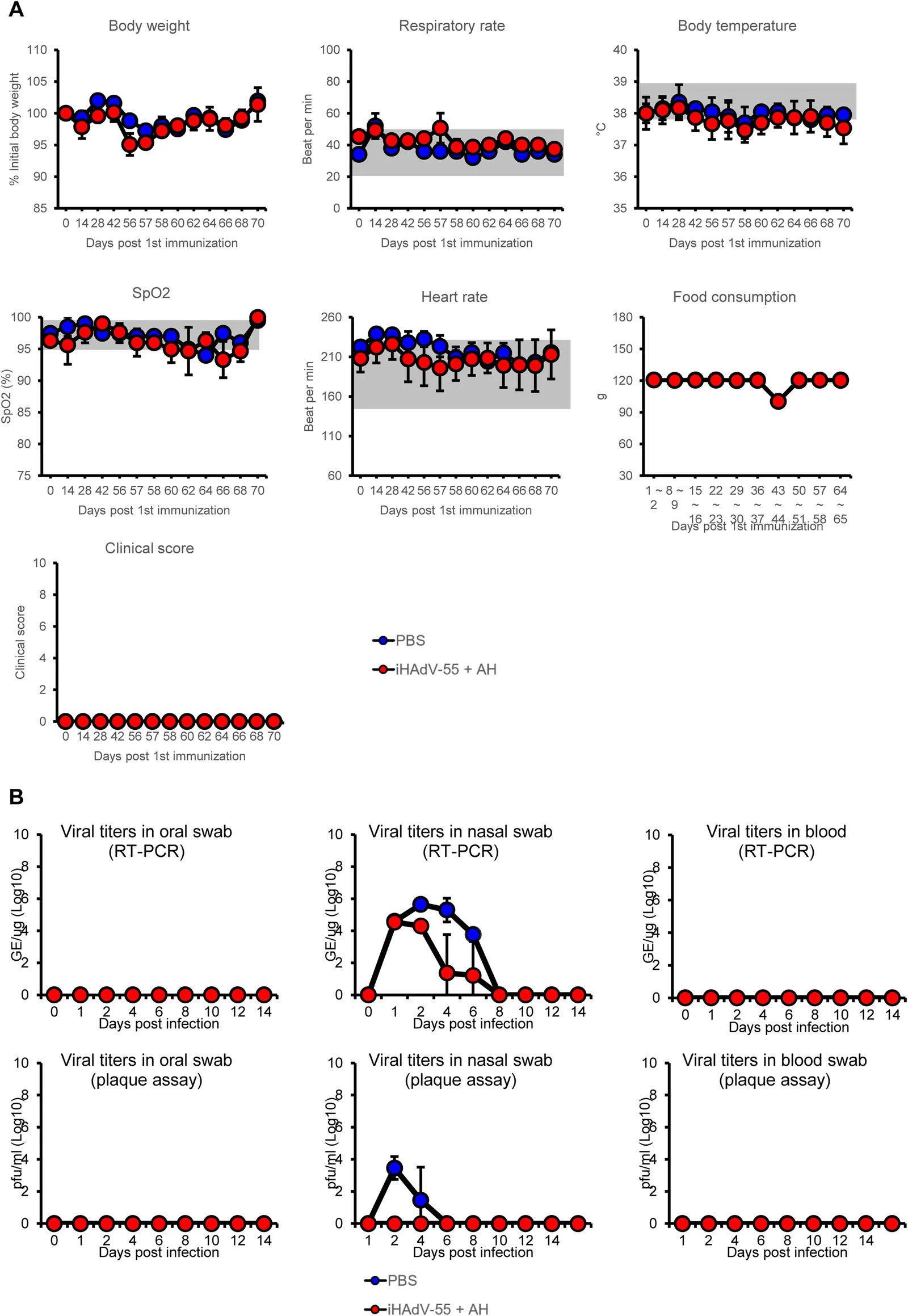
Clinical parameters and viral shedding dynamics following low dose HAdV-55 challenge. (A) Longitudinal assessment of body weight, respiratory rate, body temperature, SpO₂, heart rate, food consumption, and clinical score in PBS- and iHAdV-55 + AH-treated macaques; the x-axis is expressed as days post first immunization, with challenge performed on day 56. Shaded regions indicate reference/normal ranges as shown. (B) Viral titers in oral and nasal swabs and blood measured by RT-PCR (genome equivalents; log₁₀) and by plaque assay (PFU/mL; log₁₀); the x-axis is expressed as days post-infection (dpi), where challenge = 0 dpi (corresponding to day 56 post first immunization) and denotes the pre-challenge baseline sampling time point. Data are presented as group means with error bars as displayed.

To assess the protective efficacy of iHAdV-55 + AH under a less stringent challenge condition, clinical, physiological, and virological outcomes were evaluated following low-dose HAdV-55 infection. The clinical status of the macaques remained unremarkable post-infection. Physiological indicators, such as respiratory rate, heart rate, and SpO_2_, demonstrated minimal variability and were maintained within physiological norms throughout the observation period (Fig. 7A). Consistent with these findings, clinical scores showed no deviation from pre-challenge levels, and food intake was maintained with only a brief, minor reduction that rapidly resolved.

Virological assessment by RT-PCR detected no detectable HAdV-55 genomes in oral swabs or blood from either group (Fig. 7B). In contrast, nasal swabs showed transient viral detection during the early post-challenge period with subsequent clearance; importantly, genome copy numbers declined more rapidly in immunized macaques than in PBS-treated controls. Consistently, plaque assays identified infectious virus only in nasal swabs from PBS controls at 1–4 dpi, whereas no infectious virus was recovered from vaccinated animals, indicating that iHAdV-55 + AH immunization reduced (or prevented) shedding of replication-competent HAdV-55 after low-dose challenge.

### 3.8 Vaccination attenuates pulmonary pathology after low-dose HAdV-55 challenge

**Fig. 8:**
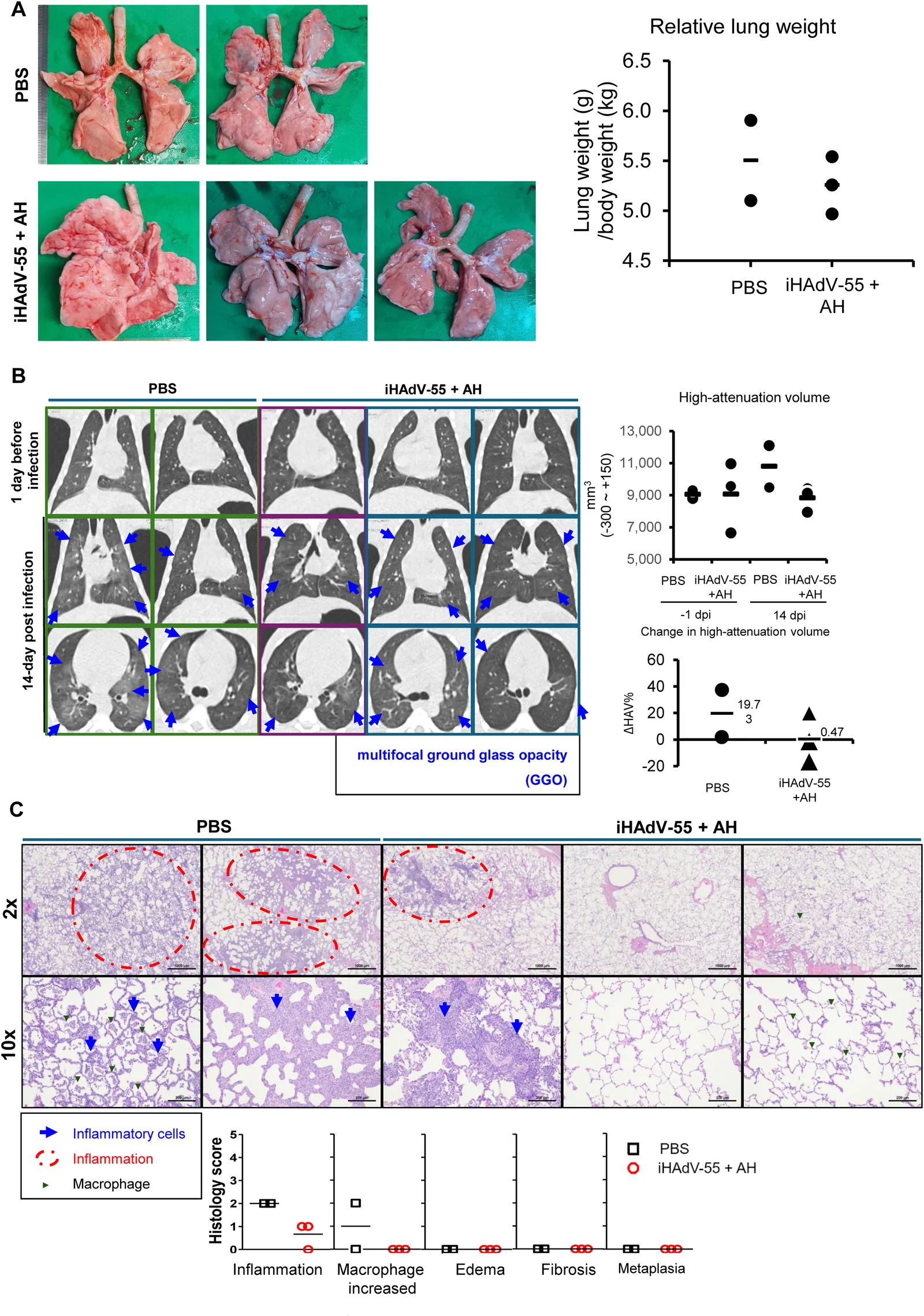
Pulmonary outcomes following low dose HAdV-55 challenge in PBS versus iHAdV-55 + AH groups. (A) Gross lung appearance at necropsy (representative images) and relative lung weight/body weight; each symbol represents an individual animal; horizontal line indicates group mean). (B) Representative CT images obtained 1 day before challenge and at 14 dpi; arrows indicate multifocal GGO. The high-attenuation volume was quantified at −1 dpi and 14 dpi. The resulting percentage change in high-attenuation volume (ΔHAV%), representing its progression of severe lesions, is depicted on the right. (C) Representative H&E-stained lung sections (2 × and 10 ×) highlighting inflammatory foci (red dashed regions), inflammatory cells (blue arrows), and macrophages (green arrowheads), with semi-quantitative histopathology scores (inflammation, increased macrophages, edema, fibrosis, metaplasia); symbols denote individual animals and horizontal lines indicate group means. HAV: high attenuation volume.

To evaluate whether the vaccine-induced protection observed in the high-dose model extends to a lower infectious burden and to determine if reduced viral shedding correlates with improved tissue-level outcomes, we performed comprehensive pulmonary assessments after low-dose (1 × 10^9^ PFU) HAdV-55 challenge. Gross and quantitative assessments indicated improved pulmonary outcomes in the iHAdV-55 + AH group compared with PBS controls, including a trend toward reduced lung involvement consistent with a lower inflammatory burden (Fig. 8A). Longitudinal chest CT imaging revealed that PBS-treated macaques developed pneumonia-associated abnormalities predominantly characterized by multifocal ground-glass opacities (GGO) (Fig. 8B). In contrast, vaccinated macaques exhibited fewer radiologic abnormalities at the same time point. The impact of vaccination on lung pathology was further assessed by longitudinal CT. At −1 dpi, HAV was comparable between groups (PBS, 9056 mm³; iHAdV-55 + AH, 9059 mm³), within the normal reference range defined above. By 14 dpi, HAV in PBS-treated animals had increased to 10,797 mm³, whereas vaccinated animals showed no net increase (8837 mm³), corresponding to a ΔHAV% of 19.7 in the PBS group and 0.5 in the vaccinated group. Consistent with the milder overall phenotype of this challenge model, individual values overlapped between groups (Supplementary Table S2b), and these differences should therefore be regarded as a trend rather than a demonstrated effect. Notably, two of three vaccinated animals showed a net decrease in HAV between the two scans (−135 mm³ and −1831 mm³), further indicating that this compartment reflects a physiological, inflation-dependent background rather than fixed pathological lesion. Because the high- and low-dose challenges were performed in separate cohorts, these values are not directly comparable with those of the high-dose study. Individual animal values are provided in Supplementary Table S2b. As in the high-dose experiment, and given the small group sizes (PBS, n = 2; iHAdV-55 + AH, n = 3), individual animal values are plotted in Fig. 8B to allow assessment of inter-animal variability, and these group-level differences should be regarded as small-sample estimates.

Histopathological evaluation further corroborated the imaging findings, providing high-resolution evidence of reduced tissue damage (Fig. 8C). Lung sections from PBS-treated controls showed evident inflammatory changes, characterized by inflammatory cell infiltration and increased macrophage-associated findings. Conversely, vaccinated animals demonstrated comparatively attenuated histologic features, indicating a preserved alveolar architecture.

Collectively, these data demonstrate that vaccination with iHAdV-55 + AH mitigates pulmonary pathology following low-dose HAdV-55 challenge, consistent with the protective trends observed under high-dose infection.

### 3.9 Immunohistochemical quantification following low-dose HAdV-55 challenge

**Fig. 9:**
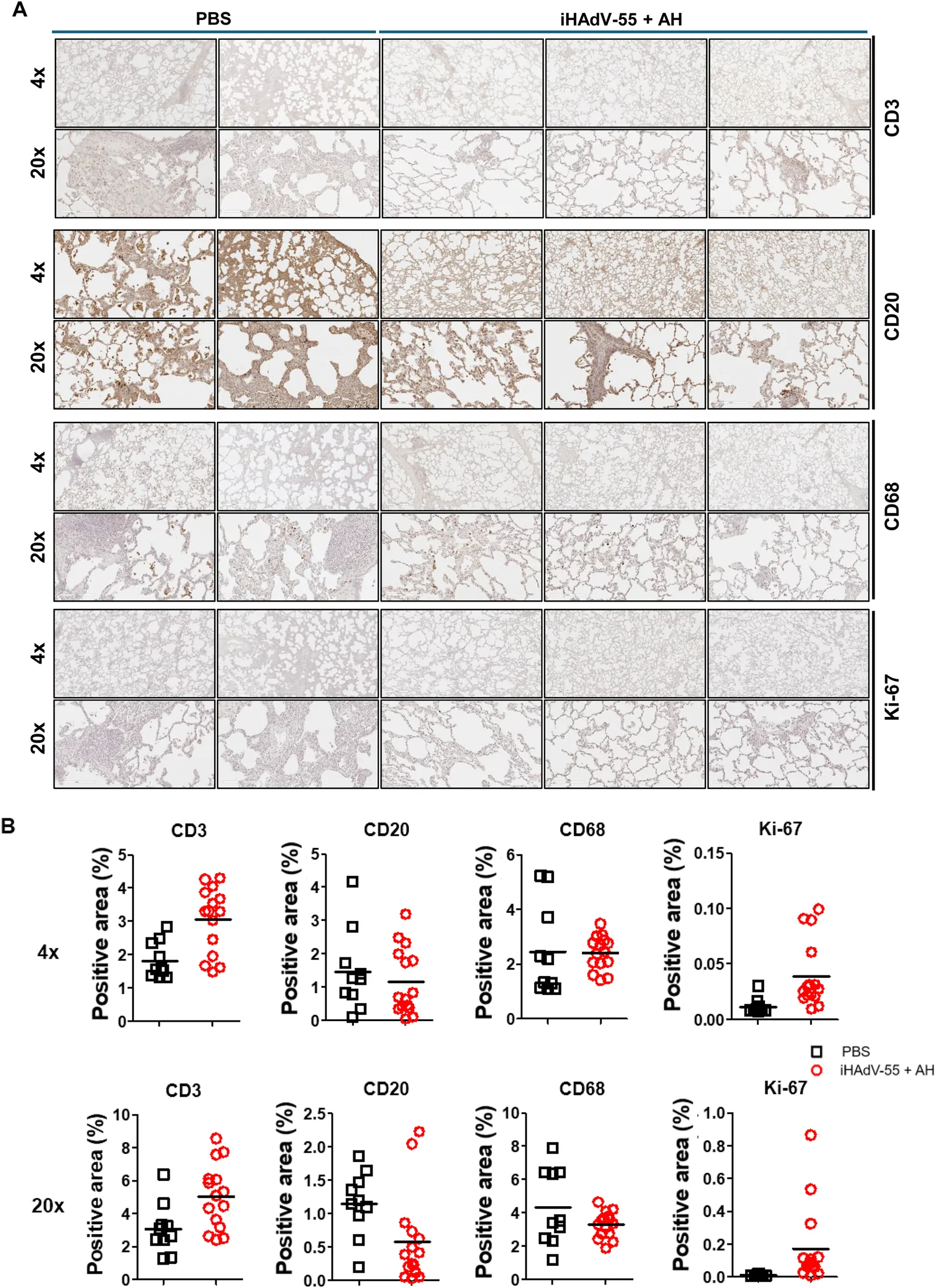
Lung immunohistochemistry after low-dose HAdV-55 challenge. (A) Representative lung sections from PBS-treated controls and iHAdV-55 + AH–immunized animals stained for CD3 (T cells), CD20 (B cells), CD68 (macrophages), and Ki-67 (proliferating cells), shown at 4 × and 20 × magnification. (B) Quantification of marker-positive area (positive area, %) at 4 × and 20 × . Each symbol represents an individual image field (PBS, black squares; iHAdV-55 + AH, red circles); horizontal bars indicate group means. Owing to the small number of animals per group (n = 2–3), no formal significance testing was performed, and differences are presented descriptively.

Following low-dose challenge, IHC profiling revealed distinct group differences in lymphocyte-associated staining and proliferative activity (Fig. 9A). The IHC staining of lung sections revealed immune cell infiltration in PBS control animals following low-dose iHAdV-55 challenge, with relatively reduced infiltrates in iHAdV-55 + AH vaccinated animals. At both 4 × and 20 × magnification, PBS lungs displayed prominent CD3⁺ T-cell and CD20⁺ B-cell aggregates primarily in peri‑bronchial and perivascular regions, appearing more diffuse and sparser in the vaccinated group. Morphometric analysis showed lower CD20⁺ positive areas in vaccinated lungs versus PBS controls at both magnifications, alongside modest CD3⁺ reductions, consistent with reduced B-cell-dominant inflammatory foci in vaccinated animals (Fig. 9B), while CD68⁺ macrophage areas remained comparable between groups. These CD3⁺ findings describe the extent of total T-cell infiltration into challenged lung tissue and should not be interpreted as a measure of antigen-specific, vaccine-elicited T-cell responses, which were not assessed in this study. Ki-67⁺ proliferating cells were more abundant in vaccinated lungs than in PBS controls; as above, the lineage of these proliferating cells was not established, and this observation is reported descriptively.

### 3.10 Associations between vaccine-elicited neutralization and pulmonary outcomes in challenge models

**Fig. 10:**
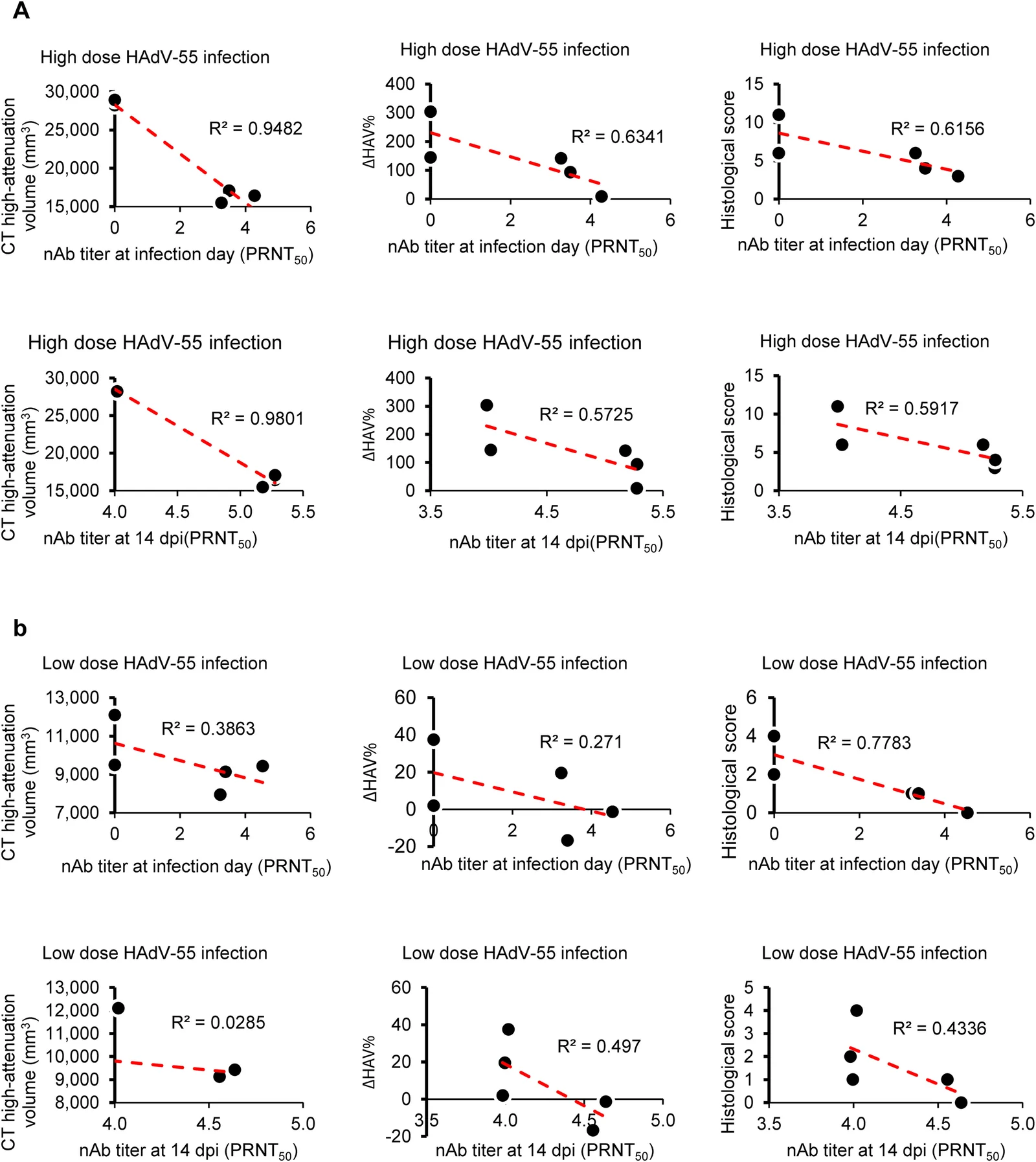
Association between HAdV-55–specific nAb titers and pulmonary disease endpoints following high- and low-dose HAdV-55 challenge. (A) High-dose HAdV-55 infection and (B) low-dose HAdV-55 infection. Scatter plots depict relationships between nAb titers measured by PRNT on the day of infection (top row) or at 14 dpi (bottom row) and pulmonary outcome measures, including CT-derived high-attenuation volume (mm^3^), ΔHAV% (as defined in Methods), and semi-quantitative lung histopathology score. Each dot represents an individual animal. Red dashed lines indicate least-squares linear regression, and the coefficient of determination (R^2^) is shown in each panel. These correlation analyses are exploratory: each panel includes only five animals, and the coefficient of determination (R²) is shown for descriptive purposes only. Given the small sample size, R² values are unstable and should not be interpreted as evidence of a defined quantitative relationship. HAV: high attenuation volume.

To generate hypotheses regarding whether neutralizing activity relates to inter-animal differences in disease severity, we performed an exploratory analysis of linear associations between HAdV-55–specific nAb titers (PRNT₅₀) and CT- and histopathology-based endpoints in the high- and low-dose challenge models (Fig. 10). We emphasize that each analysis includes only five animals; with a sample of this size, the coefficient of determination (R²) is an unstable estimator, is sensitive to individual data points, and does not constitute evidence of a quantitative correlate of protection. The R² values are therefore reported descriptively and without significance testing. Any apparent inverse trend between nAb titers and pulmonary disease burden in these plots should be regarded as preliminary and hypothesis-generating; establishing whether neutralizing antibody titer is a genuine correlate of protection will require adequately powered studies with larger cohorts.

## 4 Discussion

The global emergence of human adenovirus type 55 (HAdV-55) as a cause of severe acute respiratory disease and life-threatening pneumonia underscores the need for effective vaccine countermeasures. Using a translational cynomolgus macaque model, we evaluated the immunogenicity, durability, and protective efficacy of an alum-adjuvanted, inactivated HAdV-55 vaccine (iHAdV-55 + AH) over one year and under two respiratory challenge conditions. iHAdV-55 + AH elicited detectable and durable HAdV-55-reactive bAb and specific nAb responses and was associated with reduced pulmonary disease severity after challenge, supported by concordant CT-based pneumonia metrics and histopathology. No evidence of vaccine-associated enhanced respiratory disease was observed in either the high-dose (1 × 10¹¹ PFU) or low-dose (1 × 10⁹ PFU) setting, supporting the safety and translational promise of this inactivated vaccine approach.

Previous HAdV-55 vaccine studies have largely relied on recombinant platforms and murine readouts. A recombinant HAdV-3 expressing the HAdV-55 hexon induced nAbs and protected BALB/c mice after challenge (Liu et al., 2018; Tian et al., 2018), but wild-type mice do not naturally express key receptors implicated in HAdV-55 entry and tropism (human CD46 and desmoglein-2) (Feng et al., 2020), limiting direct extrapolation to humans. A replication-incompetent HAdV-55 candidate evaluated in human CD46-transgenic mice and Chinese macaques reduced systemic viral loads and showed passive-transfer activity (Feng et al., 2026); however, protection was not established at the primary respiratory site and key efficacy readouts remained murine system. In contrast, our study provides non-human primate (NHP) evidence that iHAdV-55 + AH attenuates lung pathology in cynomolgus macaques—a model that more closely reflects human adenoviral pneumonia (Seo et al., 2026) —and, under low-dose challenge, is associated with a reduction in recoverable infectious virus in nasal swabs of vaccinated animals.

The feasibility of a human adenovirus challenge in macaques warrants explicit consideration, since human adenoviruses can undergo restricted replication in non-natural primate hosts owing to species-specific host restriction factors. The validity of this model for our purpose rests primarily on its faithful reproduction of human-like pulmonary pathology: the cynomolgus macaque HAdV-55 model, established independently before this work, recapitulates the clinical, radiologic, histopathologic, and immunologic hallmarks of human HAdV-55 respiratory disease (Seo et al., 2026). HAdV-55 was detected in nasal swabs by both RT-PCR and plaque assay, and in the low-dose study infectious virus was recovered from control but not vaccinated animals, indicating viral persistence and shedding at the upper respiratory mucosa and a vaccine-associated reduction in recoverable infectious virus. We are cautious, however, not to overinterpret these findings as robust productive replication: because a high-titer infectious inoculum was administered and our sampling did not resolve early replication kinetics, the recovery of infectious virus in the first days after challenge cannot be cleanly separated from clearance of input virus, and the true extent of replication in this host is uncertain. The principal advantages of the model are its anatomical and immunological similarity to humans and its reproduction of pneumonia-like pathology not captured by rodent systems; its principal limitation is that HAdV-55 replication may be restricted or abortive in this non-natural host, such that some pulmonary pathology may reflect the innate response to the inoculum rather than extensive productive replication. The model is therefore best suited to assessing vaccine-mediated attenuation of pulmonary pathology and reduction of upper-airway virus, and cross-species differences in replication should be borne in mind when extrapolating to human infection.

The choice of an inactivated whole-virus platform for HAdV-55 reflects a deliberate set of trade-offs relative to the subunit and replication-incompetent vector approaches that have dominated prior HAdV-55 vaccine development. An inactivated whole-virion presents the full complement of native capsid antigens—including both hexon and fiber, which human sero-epidemiology identifies as complementary neutralization targets, with fiber-directed antibody in particular contributing cross-neutralizing activity (Feng et al., 2018). This breadth is difficult to reconstruct with a hexon-only subunit or hexon-chimeric vector immunogen (Liu et al., 2018; Tian et al., 2018) and may afford more robust type-specific neutralization. The platform is also manufacturing-mature: inactivated vaccines rely on established, scalable cell-culture and formaldehyde-inactivation processes with a well-characterized regulatory precedent, and their non-replicating nature makes them suitable for immunocompromised and other vulnerable populations who are disproportionately affected by severe adenoviral disease. Against these advantages, the inactivated platform carries recognized limitations. It is biased toward humoral immunity and, absent additional adjuvants or delivery strategies, is a comparatively weak inducer of CD8⁺ T-cell responses, which replication-incompetent vectors elicit more efficiently. Formaldehyde inactivation may also modify conformational epitopes and reduce antigenic fidelity relative to a genetically expressed subunit or vector-delivered antigen. We therefore position iHAdV-55 + AH not as inherently superior, but as a pragmatic and translationally advanced option whose demonstrated NHP efficacy and safety profile justify further development, while acknowledging that cellular and mucosal immunity remain to be optimized.

The impact of pre-existing adenovirus immunity merits specific consideration, as it distinguishes inactivated from vector-based adenovirus vaccines. For adenovirus-vectored vaccines, pre-existing vector-neutralizing antibody can neutralize the vector before transgene expression and blunt immunogenicity, a concern that is heightened when the vector and target pathogen share a serotype and that often necessitates rare or non-human serotypes (Zaiss et al., 2009; Fausther-Bovendo and Kobinger, 2014). An inactivated HAdV-55 vaccine does not rely on de novo antigen expression from an infectious particle and is therefore comparatively less vulnerable to this form of interference, a relative advantage in populations with prior adenovirus exposure. Our data are consistent with this: at baseline the macaques had detectable HAdV-55–reactive (cross-reactive, non-neutralizing) bAbs but minimal type-specific nAb, and vaccination nonetheless elicited durable type-specific nAb, indicating that pre-existing cross-reactive immunity did not preclude a de novo nAb. Nonetheless, in target populations such as military recruits, some individuals may carry genuine type-specific nAb against HAdV-55 or related species B adenoviruses (HAdV-11, HAdV-14), which could either boost or interfere with the vaccine response; because our animals were selected to be neutralization-seronegative at baseline, this cannot be assessed here and will require evaluation in previously exposed or seropositive subjects.

Testing two challenge intensities strengthened inference regarding robustness. The high-dose model served as a stringent stress test that reproducibly elicited pneumonia-like CT abnormalities in controls (patchy consolidation and multifocal ground-glass opacities) with increased lesion metrics and histologic injury. Vaccinated macaques exhibited attenuated lesion burden and lower pathology severity, indicating mitigation of clinically meaningful pulmonary pathology. The low-dose model produced a milder phenotype with limited systemic perturbations, yet pulmonary involvement remained detectable by CT and histopathology in controls; vaccine-associated improvements were again evident. Preservation of protective trends across both infectious pressures reduces the likelihood of a dose-specific artifact and supports generalizability across varying exposure intensities. We emphasize that the high-dose and low-dose challenges were conducted as separate experiments in independent animal cohorts, under conditions that were not jointly controlled. Accordingly, we interpret the concordance between the two studies only as a reproduced direction of protective effect under two independent challenge conditions, and we do not draw quantitative cross-experiment comparisons or infer a graded dose–response relationship. Numerical values from the two experiments are compared only within each experiment, against that experiment's own PBS controls.

Virologic findings further informed the likely compartment of vaccine impact. Viral detection was predominantly confined to the respiratory tract. In the low-dose study, infectious virus by plaque assay was detected in nasal swabs from PBS controls early after infection, whereas vaccinated animals showed no detectable infectious virus despite transient RT-PCR positivity, consistent with reduced shedding of replication virus in the upper respiratory tract. Because viral shedding occurs at a mucosal surface, local mucosal antibodies may contribute to this reduction in addition to systemic neutralizing antibodies. We did not measure mucosal responses (e.g., nasal IgA or bronchoalveolar lavage (BAL) antibodies), and our data therefore cannot determine whether the reduced shedding reflects a pre-existing vaccine-elicited mucosal response, an accelerated mucosal recall response induced by the challenge itself, or spillover of systemic antibody to the airway surface; these possibilities are not mutually exclusive and cannot be distinguished without direct mucosal measurement.

Mechanistically, the data support a major contribution of antibody-mediated protection. nAb titers peaked after prime–boost immunization, remained detectable through one year, and rose rapidly after challenge in vaccinated animals, whereas controls showed delayed induction. These kinetics support the interpretation that pre-existing vaccine-elicited Abs limit early viral amplification at the portal of entry and blunt downstream inflammatory cascades that drive pulmonary injury (Feng et al., 2026; Liu et al., 2024). Consistent with this model, an exploratory analysis suggested a possible inverse association between nAb titers and radiologic and histopathologic disease burden. However, because each analysis was limited to five animals, we do not interpret the high R² values in the high-dose model as evidence of a strong or defined relationship; small-sample regressions of this kind are readily influenced by individual observations and are prone to overfitting. The less consistent associations under low-dose challenge are equally compatible with the limited sample size and narrow dynamic range of pathology. These analyses should therefore be read only as a preliminary basis for formally testing neutralizing antibody as a correlate of protection in future, adequately powered studies. Additional mechanisms; the role of non-neutralizing Fc-mediated effector functions (e.g., opsonophagocytosis and antibody-dependent cellular cytotoxicity) will require targeted functional assays, mucosal measurements, and integration with virologic kinetics (Zhang et al., 2023; van Erp et al., 2019; Kim et al., 2025; Lee et al., 2025).

Immunohistochemical profiling provided complementary insight into lung tissue responses. After high-dose challenge, vaccination reduced CD20-positive area at both magnifications, while CD3 and CD68 staining were broadly comparable, suggesting limitation of B cell–rich inflammatory foci without uniform suppression of leukocyte-associated signals. Ki-67 positivity was higher in vaccinated animals. Because Ki-67 does not distinguish cell type, this signal cannot be attributed to a specific process; it may represent reparative epithelial proliferation, expansion of infiltrating immune cells, or other proliferative activity, and these possibilities cannot be separated with the present single-marker staining. Determining the cellular source will require co-staining of Ki-67 with epithelial, immune, and endothelial lineage markers, ideally by multiplex immunofluorescence. Under low-dose challenge, CD20-positive area again decreased (significant at higher magnification), CD3-positive area increased, and CD68 remained comparable, suggesting dose-dependent differences in lymphocyte-associated tissue signals that warrant higher-resolution characterization (e.g., multiplex IHC/immunofluorescence, spatial profiling, or flow cytometry of lung/BAL).

A significant limitation of this study concerns cellular immunity, which we did not characterize. The investigation was configured as a humoral-immunity and protective-efficacy evaluation, and peripheral blood mononuclear cells were not isolated or cryopreserved at any sampling time point; consequently, antigen-specific CD4⁺ and CD8⁺ T-cell responses could not be measured by interferon-gamma (IFN-γ) enzyme-linked immunospot (ELISpot) or intracellular cytokine staining, and no residual samples suitable for such analysis remain. It is important to note that the CD3⁺ T-cell signals detected by lung immunohistochemistry reflect the extent of total T-cell infiltration into challenged tissue—a downstream feature of the post-challenge inflammatory response—and are not a measure of antigen-specific, vaccine-elicited T-cell priming; the two should not be conflated. Because we did not measure antigen-specific cellular responses, our mechanistic interpretation is deliberately confined to a humoral, antibody-associated account, and we do not claim that protection was mediated by antibody to the exclusion of cellular mechanisms. This gap is particularly relevant for the platform used here: alum-adjuvanted inactivated antigens generally elicit predominantly humoral, Th2-skewed responses and are comparatively weak inducers of CD8⁺ T-cell immunity relative to replication-competent or vectored platforms, which makes direct functional measurement—rather than inference—necessary to establish whether cellular immunity contributes to the protection we observed. Dedicated antigen-specific T-cell profiling, by IFN-γ ELISpot and intracellular cytokine staining resolving the CD4⁺ and CD8⁺ compartments and complemented by memory-phenotype analysis, will therefore be an important objective for future studies of this candidate.

Several other limitations should be noted. Foremost, the group sizes (n = 2–3) were constrained by the ethical reduction mandate and the resource envelope governing non-human primate research, and were not derived from an a priori power analysis, as no prior effect-size estimate for vaccine-mediated attenuation of HAdV-55 pulmonary pathology in primates was available. In particular, the PBS control group comprised only two animals in each challenge experiment; a control arm of this size cannot capture the full range of inter-individual variation, and the between-group differences—such as the high-dose CT severe lesion volume (approximately 28,800 mm³ in controls versus 16,500 mm³ in vaccinated animals)—should be read as estimates from a small sample that may be influenced by individual variation rather than as precisely quantified effects. Consequently, this study is best regarded as a hypothesis-generating, proof-of-concept efficacy evaluation: statistical comparisons on cohorts of this size have limited power and carry a non-trivial risk of both Type I (spurious significance) and Type II error (true effects missed for lack of power) and cannot support a formal definition of correlates of protection. For this reason, we intentionally refrained from reporting formal significance tests for the immunohistochemical morphometry (Fig. 5, Fig. 9): because multiple image fields were quantified per animal, treating these fields as independent observations would constitute pseudoreplication and would overstate the true animal-level sample size, yielding statistically misleading P-values. We instead present the individual field-level measurements together with per-animal values and describe these comparisons descriptively—by the direction and consistency of the effect, most notably the reduction in CD20⁺ B-cell area in vaccinated lungs—rather than by significance thresholds. The consistency of the observed protective effects across independent high- and low-dose challenge experiments, and their concordance across orthogonal endpoints (relative lung weight, quantitative CT, and histopathology), lends internal coherence to the findings; nonetheless, adequately powered confirmatory studies with larger cohorts will be required to establish reproducibility and to formally quantify effect sizes and immune correlates before advancing this candidate. Second, post-challenge follow-up was limited to 14 days and may not capture longer-term repair or remodeling. Immune analyses focused primarily on systemic humoral responses. We did not quantify mucosal antibodies (nasal IgA or BAL antibodies), which is a particular limitation for interpreting the reduced upper-respiratory viral shedding, since mucosal antibodies—whether elicited by vaccination or induced by the challenge—could contribute directly to control of virus at the airway surface. Direct mucosal profiling will therefore be important to define the compartmental basis of the observed protection. Finally, the high- and low-dose challenges were conducted as entirely separate experiments in independent animal cohorts, with potentially differing experimental conditions. For this reason, we have refrained from direct numerical comparison of results across the two experiments, and any apparent difference between them cannot be attributed to challenge dose alone, being equally consistent with between-experiment variability. The two experiments should therefore be read as independent tests of the same hypothesis rather than as the two arms of a single dose-ranging study.

In conclusion, iHAdV-55 + AH induced durable bAb and nAb responses, attenuated pulmonary pathology, and improved post-challenge outcomes in cynomolgus macaques, with no evidence of enhanced disease. The consistency of protection across high- and low-dose challenges supports the translational potential of this inactivated vaccine strategy and highlights the utility of this NHP model for preclinical evaluation of HAdV-55 countermeasures.

## CRediT authorship contribution statement

**Jun Young Lee:** Writing – review & editing, Writing – original draft, Supervision, Methodology, Investigation, Formal analysis. **Jung-ah Choi:** Writing – review & editing, Supervision, Methodology, Investigation, Formal analysis. **Dae-Im Jung:** Writing – original draft, Methodology, Investigation, Formal analysis. **Yunjeong Park:** Writing – original draft, Methodology, Investigation, Formal analysis. **Eunji Yang:** Writing – original draft, Methodology, Investigation, Formal analysis. **Chanmi Kim:** Writing – original draft, Methodology, Investigation, Formal analysis. **Minkyung Ko:** Writing – original draft, Methodology, Investigation, Formal analysis. **Seung Ho Baek:** Writing – original draft, Methodology, Investigation, Formal analysis. **Jung Joo Hong:** Writing – review & editing, Supervision, Methodology, Investigation. **Soon-Hwan Kwon:** Writing – review & editing, Supervision, Methodology, Investigation. **Won-Tae Kim:** Writing – review & editing, Supervision, Methodology, Investigation. **Manki Song:** Writing – review & editing, Supervision, Investigation, Funding acquisition, Conceptualization. **Sang Hwan Seo:** Writing – review & editing, Writing – original draft, Supervision, Methodology, Investigation, Formal analysis, Conceptualization.

## Declaration of competing interest

The authors declare that they have no known competing financial interests or personal relationships that could have appeared to influence the work reported in this paper.

## Data Availability

Data will be made available on request.

## Appendix Supplementary materials

Image, application 1
