# Supplementary material for: An inactivated human adenovirus type 55 vaccine attenuates pulmonary pathology in a non-human primate challenge model
Source: Virus Res. 2026 Aug 21;372:199793. doi: 10.1016/j.virusres.2026.199793 (PMC13544396; doi:10.1016/j.virusres.2026.199793)
Supplement: Supplementary file 1 [file mmc1.docx]

**Supplementary Data**

An inactivated human adenovirus type 55 vaccine attenuates pulmonary pathology in a non-human primate challenge model

Jun Young Lee^a^, Jung-ah Choi^b^, Dae-Im Jung^b^, Yunjeong Park^b^, Eunji Yang^b^, Chanmi Kim^b^, Minkyung Ko^b^, Seung Ho Baek^c^, Jung Joo Hong^c,d^, Soon-Hwan Kwon^e^, Won-Tae Kim^a^, Manki Song^b*^, and Sang Hwan Seo^b*^

^a^KeyPrime Research, 193, Osongsaengmyeing 1-ro, Osong-eup, Heungdeok-gu, Cheongju-si, Chungcheongbuk-do, 28161, Republic of Korea

^b^Science Unit, International Vaccine Institute, Seoul, 08826, Republic of Korea

^c^National Primate Research Centre, Korea Research Institute of Bioscience and Biotechnology (KRIBB), Yeongudanji‑ro, Ochang‑eup, Chengwon‑gu, Cheongju, Chungcheongbuk 28116, Republic of Korea

^d^KRIBB School of Bioscience, Korea University of Science and Technology (UST), Daejeon, Republic of Korea

^e^Department of Infectious Diseases, Armed Forces Medical Research Institute, Daejeon, Republic of Korea

 **Supplementary Figure S1. Native chest CT and attenuation-compartment overlays before and after high-dose HAdV-55 challenge.** Axial and coronal images at −1 dpi and 14 dpi are shown for a representative PBS control (PBS-1) and a representative vaccinated animal (Vac-2) from the high-dose challenge study. For each time point, native CT images (lung window) are shown above the corresponding colour-coded overlays at identical slice positions. Identical Hounsfield unit windows, colour mapping and display settings were applied to all overlay panels. Attenuation compartments were displayed as superimposed layers within the segmented lung mask, using the same nominal windows as the quantitative analysis: dark green, total lung volume (TLV; −1024 to +150 HU); light green, intermediate-attenuation volume (−700 to −300 HU); brown, high-attenuation volume (HAV; −300 to +150 HU). The two narrower windows were overlaid on the total-lung layer, so that voxels remaining visible as dark green are those not covered by either narrower window, which in practice corresponds to lung below −700 HU. At −1 dpi, the native images show no consolidation or ground-glass opacity in either animal, and HAV voxels are confined to punctate and short linear structures that correspond to the pulmonary vessels, airway walls and hilar structures visible on the native images. At 14 dpi, the native images show multifocal consolidation and ground-glass opacity, and HAV voxels form confluent, area-occupying parenchymal opacities that do not follow the vascular distribution. These images demonstrate that the non-zero baseline HAV reflects normal anatomical structures and anaesthesia-related dependent atelectasis rather than pre-existing pathology.

In the PBS control, HAV increased from 11,522 mm³ (9.5% of total lung volume) at −1 dpi to 28,222 mm³ (17.7%) at 14 dpi, whereas in the vaccinated animal it increased from 8,812 mm³ (4.7%) to 17,065 mm³ (9.8%). Review of all pre-challenge scans identified no consolidation or ground-glass opacity in any of the 10 challenged animals. Representative animals were selected as the animal with the lower ΔHAV% of the two PBS controls and the animal with the median ΔHAV% of the three vaccinated animals; values for all animals are given in Supplementary Tables S1 and S2. Corresponding anatomical levels were matched visually using anatomical landmarks; exact slice-to-slice correspondence between time points was not achievable because scans were acquired in separate anaesthesia sessions under free breathing, and no image registration was applied. All quantitative measurements were derived from whole-lung three-dimensional segmentation and are therefore independent of the representative slices displayed here. dpi, days post-infection; HAV, high-attenuation volume; TLV, total lung volume.

**Supplementary Table S1. Pre-challenge (−1 dpi) quantitative computed tomography measurements in all challenged cynomolgus macaques (n = 10).** Lung masks were defined within anatomical lung boundaries and included aerated parenchyma, pulmonary vessels, airway walls and non-aerated regions; the trachea and main bronchi were excluded. All values were derived from whole-lung three-dimensional segmentation. HU windows are nominal thresholds. Because all animals were unchallenged at this time point and immunisation was administered intramuscularly, scans from PBS and vaccinated animals and from both challenge studies were pooled to define the normal reference range for this species and imaging protocol. Review of all pre-challenge scans identified no consolidation or ground-glass opacity in any animal. TLV, total lung volume; HAV, high-attenuation volume; IQR, interquartile range.

**Table S2a.**

**Table S2b.**

**Supplementary Table S2. Quantitative computed tomography measurements before and after HAdV-55 challenge.** ΔHAV = HAV at 14 dpi − HAV at −1 dpi. The high-dose and low-dose challenges were conducted as separate experiments in independent animal cohorts; values from the two studies should not be compared directly. Table S2a) High dose (1 × 10^11^ PFU), Table S2b) Low dose (1 × 10⁹ PFU) HAdV-55 challenge. TLV, total lung volume; HAV, high-attenuation volume (−300 to +150 HU); dpi, days post-infection. ΔHAV% = [(HAV at 14 dpi − HAV at −1 dpi)/HAV at −1 dpi] × 100.
